# Trifarotene: A Novel Therapeutic Option for Acne

**DOI:** 10.1155/2022/1504303

**Published:** 2022-05-27

**Authors:** Piyu Parth Naik

**Affiliations:** Department of dermatology, Saudi German hospital and clinic, Burj Al Arab, Dubai, UAE

## Abstract

Acne vulgaris, or acne, is a prevailing cutaneous predicament that customarily crops up on the face, neck, and trunk in the forms of comedones, papules, pustules, and nodules. According to epidemiologic explorations, acne affects 9.4% of the global population, making it the eighth most common disease globally. Acne perturbs up to 85% of adolescents, while it is periodically misconceived as an ailment that affects teenagers only; nonetheless, it also affects myriad adults. Acne has well-documented psychosocial ramifications, including adverse effects on self-perception, mental health, and social functioning. Trifarotene is basically a novel fourth-generation locally applied retinoid approved for the first time in the regimens of both face and truncal acnes. The exclusive topical retinoid that adheres precisely to RAR-gamma, the epidermis' most frequent isoform, is trifarotene, approved in October 2019. The current review evaluates the role of trifarotene in treating acne.

## 1. Introduction

Acne vulgaris is a pilosebaceous unit-pertinent protracted inflammatory predicament. Desquamated epithelial cells are maintained within the hair follicle due to overabundance and aberrant cohesion, culminating in ostium impedance [1]. Keratinocytes, triggered in feedback to accumulated sebum, produce proinflammatory arbitrators such as tumour necrosis factor (TNF)-*α* and interleukin (IL)-1. Following phases of remission and recurrence symbolize the clinical history. Acne can last for decades in some people and leave scars. Acne has a well-established link to depression, anxiety, and a diminished quality of life [2]. According to the Global Burden of Disease Study 2010, acne vulgaris is the eighth most common cutaneous ailment, with a global prevalence (for all ages) of 9.38 percent [3]. Pilosebaceous unit inflammation, comedo formation, and follicular hyperkeratinization, which consist of the hair follicle and the sebaceous gland that surrounds it, are the causes of acne [4]. “Retinoids are the core of topical therapy for acne because they are comedolytic, cure the antecedent microcomedone lesion, and are anti-inflammatory.” According to the AAD recommendations, they also “allow for the maintenance of clearance [5].” Retinoids influence desquamation by promoting keratinocyte differentiation and downsizing proliferation [6]. Topical retinoids restrain vital inflammatory boulevards convoluted in acne, in conjunction with leukocyte exodus, incitement of Toll-like receptors, and the catalyst Protein 1 promenade [7]. Trifarotene is the first novel fourth-generation retinoid to be accredited by the FDA in over 20 years for acne regimens. Through selective agonism of retinoic acid receptor (RAR)-gamma, the most predominant RAR isotype in the *epidermis*, trifarotene exerts more targeted, skin-specific effects than earlier generation retinoids [8]. Local application has been established to be safer, efficacious, and admissible than the vehicle in contracting inflammatory and noninflammatory acne lesions on the trunk and face [9]. Recently, a review by Cosio et al. [10] reported that trifarotene could be potentially used for nonmelanoma skin cancers, fungal infections, and photoaging in future. Trifarotene is already studied for the management of congenital ichthyosis with encouraging data on tolerability and safety. A contemporary review inspects the portrayal of trifarotene in treating acne.

### 1.1. Symptoms and Impacts of Acne

Acne patients typically present with comedones, pustules, and papules [11]. The two types of comedones are open comedones (blackheads), which are clogged follicles with apertures that expose their contents to the air, and closed comedones (white heads), which are congested follicles without an outlet [12]. Papules are snippet embossed cutaneous lesions with less than 1 cm diameter, whereas pustules are papules that have become inflammatory and filled with pus [12]. Inflammatory, swelling lesions with a diameter of at least 5 mm are known as nodules and cysts recognized in patients with severe acne [11, 12]. Acne sufferers may also have other symptoms, together with hyperpigmentation, scarring, and erythema [12].

Patients may have auxiliary detrimental repercussions in addition to the distress cognate by acne's clinical symptoms. A study elucidated that acne patients had substantially surpassing unemployment rates than controls, illustrating a concord betwixt acne and employment [13]. Acne has also been shown to sabotage social interactions [14], self-esteem, and individual's body image. It is often comorbid with psychological infirmities such as anxiety and depression [11]. Acne also has denoting ominous monetary repercussions. Consonant to one study, the annual expenditure of treating acne in Germany is over 400 million euros [15].

### 1.2. Trifarotene for Treatment of Acne

Galderma Research and Development LLC has developed trifarotene (Aklief®), a first-in-class selective retinoic acid receptor (RAR)-*γ* agonist employed to treat acne vulgaris on the trunk and face, as well as lamellar ichthyosis, under license from Mayne Pharma International Ltd. It is a fourth-generation synthetic retinoid and is effective in treating acne's primary pathogenic proceedings. On 4 October 2019, trifarotene gained its first global approval in the United States (FDA) for the topical treatment of acne vulgaris in patients of 9 years of age and older [16]. On aseptic, dry skin, once a day, in the evening, a thin layer of trifarotene 0.005 percent cream should be utilized to the diseased territories. Two pump actuations should cover the top truck, while one pump actuation should suffice to cover the face. An additional actuation for the middle and lower back can be employed [16].

A plethora of methods have been employed for the management of acne. However, there are limitations with these conventional approaches. Unsatisfactory efficaciousness, resistance of topical antibiotics, and teratogenicity with systemic isotretinoin are such examples. Laser and light-based procedures are other vital modalities for the treatment of acne vulgaris to avoid such concerns. A study by Mohamed et al. (2016) [17] investigated the clinical efficacy of intense pulsed light (IPL) to 1,064 long-pulsed neodymium : yttrium-aluminum-garnet (Nd : YAG) in treating facial acne vulgaris. Both IPL and the 1,064-nm Nd : YAG laser were found to be helpful in treating inflammatory facial acne vulgaris in the study [17].

### 1.3. Pharmacokinetics

The US FDA recently issued recommendations on the format and content of pharmacokinetic-pharmacodynamic evaluations during regulatory submissions of applications [18]. The FDA granted approval for a new drug application (NDA) for AKLIEF® (0.005 percent trifarotene) cream after following these procedures [19]. Trifarotene is the first novel retinoid medicine to be approved by the FDA in over 20 years for the treatment of acne. The applicants (Galderma in collaboration with the Certara company) used the MedermA^TM^ model in the Simcyp® population-based simulator to evaluate safe dosing information and drug-drug interactions in a pediatric population (aged 9 to 17) without the requirement for clinical trials [20]. Topical use has been shown to be safe, well tolerated, and more effective than vehicle in decreasing noninflammatory and inflammatory acne lesions on the face and trunk [9]. The FDA granted topical trifarotene orphan drug designation for the treatment of congenital ichthyosis in 2014 after it was shown to be safe and well tolerated [21].

In ex vivo human keratinocytes, trifarotene is eminently metabolically durable, yet it is expeditiously metabolized by hepatic microsomes, culminating in considerable cutaneous commotion while conforming systemic concentrations are meager, according to Aubert and coworkers [22]. Other retinoids, such as adapalene (>60 minutes) and tazarotenic acid (57 minutes), have immense protracted hepatic microsomal half-lives. When treating enormous fractions of the body, such as truncal acne, the endowment of trifarotene to be briskly expunged by the liver is pivotal, as it warrants for the attrition of safety matters affiliated with systemic penetration [22, 23].

Trifarotene plasma concentrations were only quantifiable in 18 percent of children and 37 percent of adults who used the 50 *μ*g/g formulation vs. 61 percent of adults and children who drew on the 50 *μ*g/g formulation in two clinical studies of pharmacokinetics and safeness in pediatric (10–17 years) and adult (18–34 years) moderate-to-severe acne vulgaris cases [24]. Trifarotene 50 *μ*g/g had a lower systemic absorption than trifarotene 100 *μ*g/g. Trifarotene had a short half-life (2 to 9 hours) and did not hoard systemically despite periodic administrations. The majority of adverse events (AEs) in the 100 *μ*g/g group were cutaneous and marginally aggrandized. There were no hematologic and biochemical abnormalities found. Trifarotene is well tolerated systemically and locally, as well as safe in both adults and children, according to the findings of this study. Given that the target patient population for trifarotene comprises women of reproductive age and that the retinoid medication league has been linked to teratogenicity [25], a healthy volunteer drug-drug interaction research was conducted to descry how trifarotene interacts with oral contraceptives [24]. Patients were given a levonorgestrel 0.15 mg/ethinyl estradiol 0.03 mg (LNG/EE) combination oral contraceptive, as well as trifarotene 100 *μ*g/g. Trifarotene had no leverage on plasma grades of LNG/EE, and there were no clinically significant drug-drug interactions, suggesting that the predetermined perks of oral contraceptives are sustained even when trifarotene is exercised.

Trifarotene is excreted in feces, and due to RAR-*γ* selectivity, it can be presumed that trifarotene is safer than other previous retinoids in pregnancy [10].

### 1.4. Therapeutic Trials

#### 1.4.1. Longer-Term Phase III Trial

A trial by Blume-Peytavi et al. [26] evaluated the long-term safety and efficacy of trifarotene in both facial and truncal acne. In patients aged 9 years or older with moderate facial and truncal acne, the encouraging effects of topical trifarotene were ascertained and acne ameliorated over the course of the 52-week research duration [26]. IGA success rates were 26.6 percent, 43.3 percent, 50.1 percent, 57.6 percent, and 65.1 percent at weeks 12, 20, 26, 38, and 52, respectively, with PGA success rates of 38.6 percent, 54.1 percent, 58.4 percent, 62.5 percent, and 66.9 percent. At 52 weeks, the overall success rate was 57.9%. Most evaluable patients reported some improvement in health-related QOL (HR-QOL) at 52 weeks, according to the Children Dermatology Life Quality Index (C-DLQI; patients aged 16 years at baseline; *n* = 246 and 177) and Dermatology Life Quality Index (DLQI; patients aged 17 years; *n* = 208 and 171 evaluable at baseline and week 52) scores. At 52 weeks, 53.8 percent of adults asserted that acne had no effect on HR-QOL, and 35.7 percent said it had a minor effect. At 52 weeks, 54.2 percent of pediatric patients reported no effect or a modest effect of acne on HR-QOL, whereas 40.1 percent proclaimed nil impact [26]. If tolerability adversities were identified, the investigator curtailed the application frequency of trifarotene and, depending on IGA and PGA grading, therapies at the face and/or truncal region could be discontinued. At baseline, the safety population (*n* = 453) had an average age of 18.3 years, a male-to-female ratio of 50.1 percent, and mean counts of face inflammatory, facial noninflammatory, truncal inflammatory, and truncal noninflammatory lesions of 36.9, 58.2, 43.4, and 56.1, respectively [24]. Assorted executed and evolving clinical trials of trifarotene are delineated in Table 1.

#### 1.4.2. Pivotal PERFECT Trials

A study by Tan et al. [9] assessed the safety and efficacy of trifarotene 50 µg/g cream, a novel topical retinoid, in moderate facial and truncal acne. The eligibility criteria of the patients were age 9 years and older, moderate facial acne according to AGA score. Two phase III double-blind, randomized, vehicle-controlled, 12-week studies of once-daily trifarotene cream (0.005) vs. vehicle in subjects aged 9 years or older [9]. The trifarotene group had higher IGA success rates than the vehicle group, as did improvements in noninflammatory lesion counts and inflammatory lesion counts. IGA success rates endorsed trifarotene over vehicle therapy as early as 4 weeks in both studies, and investigator-assessed mean abatements in noninflammatory and inflammatory lesion counts advocated trifarotene from 2 weeks onwards. In these phase III trials, IGA and Physician's Global Assessment (PGA) fruition was defined as at least a 2-grade improvement and an IGA/PGA rating of clear (0) or almost clear (1). The average age of patients in the ITT population was 19.6 years, 45 percent were males, and the average number of truncal noninflammatory lesions was 46.4, facial noninflammatory was 52.1, facial inflammatory was 35.7, and truncal inflammatory was 37.2 [9].

### 1.5. Ongoing and Future Studies

There are two concluded trials whose outcomes have yet to be published. The first study evaluated the outcomes with the use of trifarotene 50 *μ*g/g cream in the treatment of moderate facial and truncal acne vulgaris. The participant had moderate acne at screening and baseline was included in this study. For 24 weeks, participants used trifarotene 50 g/g topically once daily in the evening [27]. The second trial assessed the efficacy and safety of different concentrations (25 *μ*g/g, 50 *μ*g/g, and 100 *μ*g/g) of CD5789 cream in participants with acne vulgaris for the purpose of dose identification for 12 weeks. The results were compared with those of placebo [28]. A randomized, double-blind, placebo-controlled research compared the efficacy and safety of trifarotene (cd5789) cream vs. an oral antibiotic for the treatment of severe acne vulgaris. Participants with a clinical diagnosis of acne vulgaris, defined by an IGA score of 4 (severe), were included in this study. For 12 weeks, the experiment group will apply trifarotene 50 mcg/g cream topically to the face once daily in the evening and will take one doxycycline hyclate delayed-release 120 mg tablet orally in the evening on days 1, 2, and 3, and one tablet in the morning on Day 2. The control group will receive one tablet of placebo doxycycline hyclate delayed-release 120 mg orally in the evening on days 1, 2, and 3 for 12 weeks, as well as one tablet in the morning on Day 2 [29].

### 1.6. Adverse Events

Locally applied trifarotene was generally well tolerated in pivotal and long-term phase III trials in adults aged 9 years and older with moderate facial and truncal acne vulgaris [9, 26]. In 12-week phase III trials, the majority of treatment-emergent adverse events (TEAEs) were mild to moderate skin administration spot manifestations, with only a small number of patients terminating therapy as a consequence of these aftermath [9]. In a pooled analysis of the 12-week studies (*n* = 1200/group), sunburn (2.6 vs 0.5 percent), irritation of the application site (7.5 vs 0.3 percent), and pruritus of the application site (2.4 vs 0.8 percent) were all reported in 1% of trifarotene recipients and at an inflated incidence than with vehicle treatment [16]. After longer-term treatment, the pattern of adverse events was similar to that found in pivotal studies, with 2.9 percent discontinuing treatment due to these occurrences and 12.6 percent of trifarotene patients experiencing at least one adverse reaction. In this 52-week experiment, the frequency of adverse responses deflated over time, with irritation of the application site (4.2 percent), itching over administration location (4.6 percent), and sunburn being the most common (5.5 percent) [16]. During pivotal studies, the majority of local skin irritation events were mild to moderate in intensity and were consistent with the documented tolerability profile of topical retinoid treatments in trifarotene recipients [9]. Up to 30% of trifarotene recipients had a worst postbaseline score of moderate for individual signs and symptoms on the face in pivotal 12-week trials, with a worst score of severe occurring in up to 6% of trifarotene recipients on the face; rates of truncal acne with a worst postbaseline score of severe or moderate were up to 5% and up to 19%, respectively. The highest severity scores for facial and truncal acne transpired at weeks 1 and 2–4, respectively, after which time scores improved [16]. The 1-year phase III trial demonstrated that trifarotene had a similar local tolerability profile to the pivotal 12-week phase III trials [16].

Because of the pharmacologic class of trifarotene and the potential teratogenicity of the class, as well as the fact that trifarotene is intended for use by women of childbearing potential, a drug-drug interaction study was conducted to assess the potential effects of trifarotene on contraceptive steroids, as recommended by European Medicines Agency guidelines [30]. Preclinical studies showed that trifarotene had a large safety margin for teratogenic effects and no in vitro drug-drug interaction potential; however, a clinical drug-drug interaction study was conducted to provide clear guidance in terms of risk mitigation measures by specifically addressing possible interactions affecting the efficacy of levonorgestrel/ethinyl estradiol (LNG/EE, Portia, Teva Pharmaceuticals USA, Inc, Forest, and Virginia). LNG/EE is a well-known hormonal oral contraceptive that has been on the market since the early 1970s [24].

Patient assessments and investigator-initiated long-term studies have demonstrated constant enhancement regarding trifarotene and its high-selectivity has contributed to a preferable local tolerability profile than earlier retinoids [8].

### 1.7. Cost Utility of Trifarotene

Trifarotene has a significant cost disadvantage, with a 45 g pump costing more than 500 US dollars [31]. The pharmaceutical company that developed trifarotene offers a discount programme for commercially insured and uninsured patients, allowing them to purchase a 45-g pump for 0 and 75 US dollars, respectively. Pricing is based on state-based prescription. This discount programme, however, is not available to patients enrolled in government-run or government-sponsored health care plans [32]. A 45 g container of adapalene 0.1 percent gel and a 30 g tube of tazarotene 0.1 percent cream, for example, cost roughly 34 and 47 US dollars, respectively, but a 30 g tube of tazarotene 0.1 percent cream costs around $68 [33]. Despite the manufacturer's price reductions for qualifying patients, treatment may still be out of reach for many. Furthermore, trifarotene is now only available via prescription, although adapalene 0.1 percent gel, a retinoid, is available over-the-counter [23].

### 1.8. Current Status of Trifarotene in Acne

As research is still evolving in the case of trifarotene, data continue to pour from all over the world. However, trifarotene has been accepted well by patient groups and dermatologists globally. The key indication of trifarotene is truncal acne and dermatologists can select this agent in patients more than 9 years of age. Truncal acne patients who are in dire need of rapid response are ideal candidates of trifarotene [34].

Dermatologists should clearly explain to the patient at the onset of treatment about required care with trifarotene. Erythema, dryness, and flaking are expected in few cases. However, adequate spacing between application periods and the use of moisturizer can take care of these unwanted effects [34].

It is of the utmost importance to educate the patient on the steps of trifarotene application. First, areas to be treated should be cleaned with a mild or soapless cleanser and pat dry. Next, a thin layer of trifarotene cream should be applied in the evening [16]. In order to maintain uniformity, one pump for the face and two pumps for the chest, shoulders, and back are recommended [16]. Moisturizer can be applied as often as needed to minimize dryness and irritation. Avoiding sun is also endorsed [16]. In clinical trials till date, trifarotene has shown excellent efficacy and potency. Nevertheless, many trials are still ongoing, and the strategy to manage nonresponding cases is still evolving. Ideally, patients who do not respond to trifarotene would require reassessment with a dermatologist and further evaluation.

## 2. Conclusion

Acne vulgaris is a prevailing cutaneous ailment that has a detrimental impact on one's quality of life and self-esteem. Trifarotene, a first-in-class fourth-generation locally applied retinoid, has been approved by the FDA for the treatment of acne vulgaris. It is the first topical retinoid that has been explored precisely in both facial and truncal acnes. Trifarotene has a strong selectivity for the skin-predominant RAR-gamma, implying a better local tolerability profile than older generation retinoids, which bind to the other RAR subtypes nonselectively. This hypothesis will be investigated in direct comparative studies and data on trifarotene efficacy compared to other topical retinoids. Assorted, executed, and evolving clinical trials have testified that trifarotene has excellent tolerability and safety. An accurate local application amount is critical to avoid administration site adverse effects. In culmination, trifarotene is certainly an innovative game-changer in the age-old battle against the acne.

## Figures and Tables

**Table 1:** Important clinical trials of trifarotene

Drug(s)	Location(s)	Indication	Phase	Identifier	Sponsor	Status
Trifarotene cream vs vehicle cream	France, Germany, Spain	Lamellar ichthyosis	II	NCT03738800; EudraCT 2018-003272-12; 18-ICH-001	Mayne Pharma International Ltd.	Ongoing
Trifarotene cream	USA	Early cutaneous T-cell lymphoma	I	NCT01804335; 2012–0710; NCI-2014-01377	MD Anderson Centre; Galderma R&D	Completed
Trifarotene cream vs vehicle cream and tazarotene	USA	Acne vulgaris	II	NCT01616654; RD.06. SPR18223	Galderma R&D	Completed
Trifarotene cream	USA	Acne vulgaris	III	NCT03915860; RD.06. SPR.118295	Galderma R&D	Ongoing
Trifarotene cream	Multinational	Acne vulgaris	III	NCT02189629; EudraCT 2014-001755-23	Galderma R&D	Completed
Trifarotene cream vs vehicle cream	Multinational	Acne vulgaris	III	PERFECT 2; NCT02556788; EudraCT 2016-002540- 13	Galderma R&D	Completed
Trifarotene cream vs vehicle cream	Multinational	Acne vulgaris	III	PERFECT 1; NCT025566369; EudraCT 2016-002860- 15	Galderma Research and Development (R&D)	Completed

## Data Availability

Data sharing is not applicable to this article as no datasets were generated during the current study.
